# Quantification of Silicon in Rice Based on an Electrochemical Sensor via an Amplified Electrocatalytic Strategy

**DOI:** 10.3390/mi12091048

**Published:** 2021-08-30

**Authors:** Li Fu, Yuhong Zheng, Pengchong Zhang, Guosong Lai

**Affiliations:** 1 Key Laboratory of Novel Materials for Sensor of Zhejiang Province, College of Materials and Environmental Engineering, Hangzhou Dianzi University, Hangzhou 310018, China; 2 Institute of Botany, Jiangsu Province & Chinese Academy of Sciences (Nanjing Botanical Garden Mem. Sun Yat-Sen), Nanjing 210014, China; zhengyuhong@cnbg.net; 3 Hangzhou Botanical Garden (Hangzhou West Lake Research Institute of Garden Science), Hangzhou 310013, China; zhang-pengchong@163.com; 4 Hubei Key Laboratory of Pollutant Analysis & Reuse Technology, Department of Chemistry, Hubei Normal University, Huangshi 435002, China; gslai@hbnu.edu.cn

**Keywords:** silicon content, electrocatalytic reduction, HF extraction, rice tissue, electrochemical sensor

## Abstract

Silicon plays a very important role in the growth of rice. The study of the relationship between rice and silicon has become a hot area in the last decade. Currently, the silica-molybdenum blue spectrophotometric method is mostly used for the determination of silicon content in rice. However, the results of this method vary greatly due to the different choices of reducing agents, measurement wavelengths and color development times. In this work, we present for the first time an electrochemical sensor for the detection of silicon content in rice. This electrochemical analysis technique not only provides an alternative detection strategy, but also, due to the rapid detection by electrochemical methods and the miniaturization of the instrument, it is suitable for field testing. Methodological construction using electrochemical techniques is a key objective. The silicon in rice was extracted by HF and becomes silica after pH adjustment. The silica was then immobilized onto the glassy carbon surface. These silica nanoparticles provided additional specific surface area for adsorption of sodium borohydride and Ag ions, which in turn formed Ag nanoparticles to fabricate an electrochemical sensor. The proposed electrochemical sensor can be used for indirect measurements of 10–400 mg/L of SiO_2_, and thus, the method can measure 4.67–186.8 mg/g of silicon. The electrochemical sensor can be used to be comparable with the conventional silicon-molybdenum blue spectrophotometric method. The RSD of the current value was only 3.4% for five sensors. In practical use, 200 samples of glume, leaf, leaf sheath and culm were tested. The results showed that glume had the highest silicon content and culm had the lowest silicon content. The linear correlation coefficients for glume, leaf, leaf sheath and culm were 0.9841, 0.9907, 0.9894 and 0.993, respectively.

## 1. Introduction

Silicon is one of the beneficial elements for all plants and is one of the essential nutrients for species of the Poaceae (rice, wheat, etc.), *Beta*, *Equisetum* and some diatom species [1,2,3]. Silicon is a component of plant cell walls, involved in the synthesis and transport of plant carbohydrates, and has an important impact on plant photosynthesis and transpiration [4]. When plants are deficient in silicon, they often show symptoms such as dwarf plants, slow growth and susceptibility to diseases [5]. On the other hand, silicon is also closely related to human health and is one of the essential trace elements in the human body [6]. In the human body, silicon is involved in the process of bone calcification, helps connective tissue cells to form extracellular cartilage matrix and maintains normal cardiovascular function. Silicon deficiency in the human body can affect the normal development of bones and lead to coronary heart disease [7]. The human body cannot synthesize silicon itself but must obtain it through the intake of food. A daily intake of 20 to 50 mg of silicon from food is sufficient for the normal growth and development of the human body [8].

As the fourth most important nutrient for rice, silicon has an important impact on rice growth and quality [9]. The application of silicon fertilizer on rice has become a hot research topic in the field of rice nutrient management [10,11,12]. In contrast, the methods for the detection of silicon content in plants have not been updated. Currently, silica-molybdenum blue spectrophotometric method is mostly used for the determination of silicon content in rice [13]. However, the results of this method varied greatly due to the different choices of reducing agents, measurement wavelengths and color development times [14]. For example, Wang et al. [15] used ascorbic acid as the reductive, and determination is conducted at 810 nm. Okorie et al. [16] used a similar reducing agent but determined it at 825 nm. In addition, there are various methods for pretreatment of rice samples, such as weight method [17], alkali-oxygen digestion [18], high-temperature alkali fusion method [19], digestion method [20] and autoclaving method [21]. These pretreatment techniques can also affect the results of the detection results. Electrochemical analysis technique is a branch of analytical chemistry. This analytical technique has become a suitable analytical technique for field testing due to its speed, sensitivity and miniaturization of instruments [22,23,24,25,26,27,28,29]. Electrochemical analysis techniques are not suitable for the detection of silicon because silicon is highly stable and does not participate in significant electrochemical reactions at the electrode surface. Therefore, silicon is often used as an excellent substrate for catalyst immobilization. There is a direct correlation between the performance of the catalyst and the amount of substrate, so the signal of the catalytic reaction can reflect the amount of substrate. This gives a strategy that can be used for the indirect determination of silicon. The silicon content can be measured by the amount of performance of the immobilized catalyst. In this work, we proposed for the first time an electrochemical sensor for the determination of silicon content in rice. First, the silicon in the rice tissue was dissolved using hydrochloric and hydrofluoric acids. Then, calcium ions were added to the solution to precipitate fluoride ions, resulting in soluble silica in the solution. This silica was immobilized onto the surface of glassy carbon electrode. An electrochemical sensor was then fabricated via a dipping process for a rapid deposition of small-sized silver nanoparticles. The electrochemical signals recorded on the sensor with and without silica toward hydrogen peroxide reflect the silicon content in rice. This electrochemical-based sensing strategy is a profiling approach to silicon determination which is used not only for the determination of silicon content in plants. In addition, using the performance of the catalyst to reflect the amount of inert substrates provides a new analytical methodology. This methodology can be further extended to the measurement of other electrochemically inert nanoparticles.

## 2. Materials and Methods

### 2.1. Reagent and Samples

All reagents used in this work were analytical grade. Mili-Q water has been used for all solution preparation. Glume, leaf, leaf sheath and culm of rice have been used as real samples for silicon content determination. All plant tissues were ground into powder to pass a 60-mesh screen and then dried at 80 °C for 24 h.

### 2.2. Sample Pre-treatment

10 mL of 2 M HF were added into 1 g dried plant tissue powder and shaken on a rotary shaker at 200 rpm overnight for 6 h. Then, 1 M CaCl_2_ was added to the slurry until no white precipitate was produced. Then, HCl (0.5 M) was used to adjust the pH to 2.0. Finally, the slurry was filtered using filter paper. The filtrate contains the soluble silica was dispersed to form 500 mL and used for electrode modification and silicon-molybdenum blue spectrophotometric detection.

### 2.3. Electrochemical Sensor Preparation

A certain amount of the above filtrate or standard SiO_2_ solution was drop-coated on the surface of a glassy carbon electrode (GCE) and dried at room temperature (denoted as Si/GCE). Then, the modified GCE was dipped into an NaBH_4_ solution (0.5 M) for 10 s, followed by an air blow to remove any excess liquid. GCE was then dipped into an AgNO_3_ solution (10 mM) for 10 s and dried by air blower to form a designed electrochemical sensor. The prepared electrochemical sensor was denoted as Si/Ag/GCE. SEM images (Zeiss-EM10C-100 KV, Germany) of GCE, Si/GCE and Si/Ag/GCE were shown in Figure S1. The electrochemical sensor without SiO_2_ was prepared using a similar process without the drop coating of filtrate and denoted as Ag/GCE.

### 2.4. Electrochemical Measurement

All electrochemical measurements were conducted using a CHI760e working station (Chenhua Instrument Co., Ltd., Shanghai, China). The above prepared electrode was used as a working electrode. An Ag/AgCl (3 M) and Pt wire were used as reference electrode and counter electrode, respectively. Cyclic voltammetry (CV) and linear sweep voltammetry (LSV) have been used for measuring the electrocatalytic reduction of hydrogen peroxide. The electrolyte is a 0.1 M phosphate buffer solution (PBS).

### 2.5. Silicon-molybdenum Blue Spectrophotometric Assay for Silicon Content Detection

The sample powder was added to a Teflon beaker, and 10 mL of the digestion solution (HNO_3_:HClO_4_= 9:1, *v*/*v*) was slowly added and soaked overnight to give a reddish-brown solution. Then, 5 mL of H_2_O_2_ was added to the slurry and heated to 200 °C. After cooling to room temperature, 4 mL of HNO_3_ was added and reheated to 200 °C. When a gelatinous precipitate appeared, 30 mL of water was added. 3 mL of 50% NaOH solution was added and kept at 120 °C for 40 min when the solution turned to light yellow. 2.7 mL of water, 200 μL of the above solution, 1.5 mL of 0.26 M HCl and 200 μL of 10% ammonium molybdate solution were added into a 10 mL centrifuge tube in order. The solution was shaken and allowed to stand for 5 min. Then, 200 μL of 20% tartaric acid and 200 μL of 2% ascorbic acid were added. The solution was shaken well and allowed to stand for 25 min, then it was measured colorimetrically at 600 nm.

## 3. Results and Discussion

**Figure 1:**
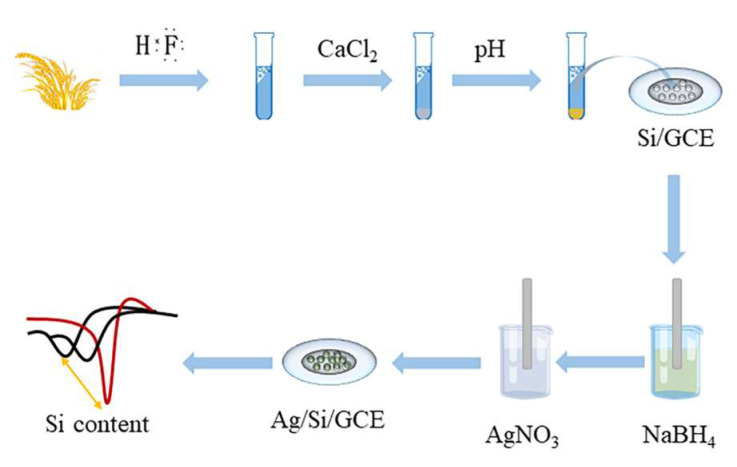
Schematic diagram of electrochemical sensor fabrication.

Figure 1 shows our proposed electrochemical sensor fabrication. First, silicon in rice tissue was dissolved in HF solution. By adding HCl to pH = 1–2, the fluorosilicate will hydrolyze and produce soluble silica. The addition of calcium chloride then removes excess fluoride ions, ensuring safety during analysis. A solution containing soluble silica was drop coated to the surface of the GCE. The silica nanoparticles were formed after the solvent evaporation. These silica nanoparticles can increase the specific surface area of GCE and be used to adsorb sodium borohydride during the dipping process. GCE containing silica was immersed into an NaBH_4_ solution. In this process, NaBH_4_ can be adsorbed on the surface of the electrode. Then, when the electrode was immersed into AgNO_3_, Ag ions were generated on the surface of the electrode due to the reducing properties of NaBH_4_ and form a designed electrochemical sensor. The silica nanoparticles on the sensor surface provide additional growth sites for Ag nanoparticles. Thus, the GCE modified with silica can grow extra Ag nanoparticles. This simple dipping reduction method was proposed by our previous works for electrochemical sensor fabrication that can be successfully used to synthesize nanocomposites on the sensor surface [30,31]. Ag nanoparticle electrocatalysis of hydrogen peroxide is a widely known reaction. The performance of electrocatalysis tends to be positively correlated with the number of Ag nanoparticles on the sensor surface. Therefore, if we compare the electrode with Ag nanoparticles grown directly on GCE and Ag nanoparticles grown on GCE after silica modification, we can know the catalytic effect of Ag nanoparticles grown on silica on hydrogen peroxide. This difference in catalytic effect recorded on the electrochemical sensor can reflect the amount of silica nanoparticles and therefore corresponds to the amount of silicon content in the rice tissue.

**Figure 2:**
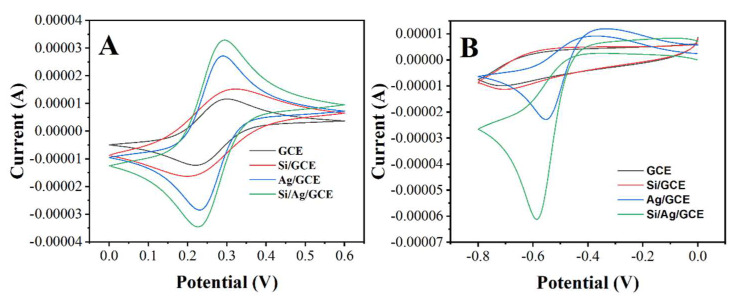
(**A**) CV profiles of GCE, Si/GCE, Ag/GCE and Ag/Si/GCE in 5 mM [Fe(CN)_6_]^3−/4−^. (**B**) CV profiles of GCE, Si/GCE, Ag/GCE and Ag/Si/GCE in 0.1 M PBS with 0.5 mM H_2_O_2_ (pH = 7.0).

To determine the feasibility of the proposed electrochemical sensor, we first used a 50 mg/L solution of soluble silica for the modification of GCE. Figure 2A shows the CV profiles of bare GCE, Si/GCE, Ag/GCE and Ag/Si/GCE in potassium ferricyanide/potassium ferricyanide. It can be seen that the modification of GCE by SiO_2_ increases the specific surface area of the sensor surface because the area of CV increased after the modification [32]. However, the peak-to-peak separation becomes larger, which is due to the semiconducting property of silica, which slows the electron transfer efficiency [33]. On the contrary, the GCE modified with Ag nanoparticles has a very high current, while the peak-to-peak separation becomes smaller due to the excellent conductivity of the Ag nanoparticles [34]. Ag/Si/GCE exhibits the largest specific surface area and also has good electron transfer performance.

The CV curves in Figure 2B show the electrochemical reduction of 0.5 mM H_2_O_2_ on the electrochemical sensors with different modifications. It can be seen from the figure that GCE can reduce H_2_O_2_ at −0.7 V, which is in good agreement with previous reports [35,36,37]. A very similar electrochemical response can also be observed in Si/GCE. Although SiO_2_ increases the specific surface area of the sensor, the current of the reduction peak does not increase substantially because the electrochemical reduction of H_2_O_2_ is not achieved on the SiO_2_ surface. On the contrary, Ag/GCE presents a huge electrochemical reduction peak at −0.59 V, corresponding to the electrocatalytic reduction of H_2_O_2_. This increase in current and the negative shift in overpotential is due to the excellent electrocatalytic properties of Ag nanoparticles [38,39]. Ag/Si/GCE exhibited an electrochemical behavior very similar to that of Ag/GCE. A reduction peak can be observed around −0.57 V. The current of the reduction peak was larger on Ag/Si/GCE than on Ag/GCE, suggesting that more Ag nanoparticles can be modified to the sensor surface due to the presence of SiO_2_ nanoparticles, which can then be involved in the electrochemical catalytic reduction of H_2_O_2_. Therefore, comparing the current difference on Ag/Si/GCE and Ag/GCE can be used as an indicator for the amount of SiO_2_ on the sensor surface.

**Figure 3:**
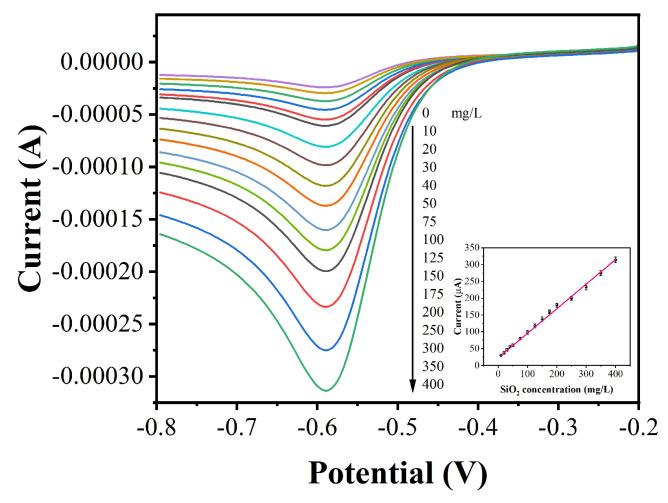
LSV profiles of Ag/Si/GCEs prepared using 10–400 mg/L of SiO_2_ for electrocatalytic reduction of 0.5 mM H_2_O_2_ in 0.1 M PBS (pH = 7.0).

In order to reflect the SiO_2_ content with the proposed electrochemical sensor, 10–400 mg/L silica solution and prepared Ag/Si/GCEs were used for 0.5 mM H_2_O_2_ reduction. Figure 3 shows the corresponding LSV curves, which show that the current value of electrochemical catalytic reduction of H_2_O_2_ and the concentration of silica solution were linearly related. This is because the signal of electrochemical catalysis is related to the number of Ag nanoparticles [40]. Moreover, the number of Ag nanoparticles is related to the number of Si deposited on the GCE. Therefore, the proposed electrochemical sensor can be used for indirect measurements of 10–400 mg/L of SiO_2_, and thus, the method can measure 4.67–186.8 mg/g of silicon.

To verify the accuracy of the proposed electrochemical sensor, the sensor was tested using a standard addition recovery protocol and compared with the silicon-molybdenum blue spectrophotometric method. Silicon standard solutions were used for silicon-molybdenum blue spectrophotometric method, while SiO_2_ dispersion was used for the proposed electrochemical method (calculated Si content has been used in Table 1). The results were shown in Table 1. It can be seen that the proposed electrochemical sensor can be used to be comparable with the conventional silicon-molybdenum blue spectrophotometric method. The stability and repeatability of the sensor are important properties. We tested five prepared Ag/Si/GCEs and they showed excellent performance for the electro-catalytic reduction of 0.5 mM H_2_O_2_. The RSD of the current value was only 3.4%. We also performed six consecutive tests on a single Ag/Si/GCE. Since the reduction reaction of H_2_O_2_ does not produce products that poison the electrode [41], there is no significant difference in the current values after six tests.

**Figure 4:**
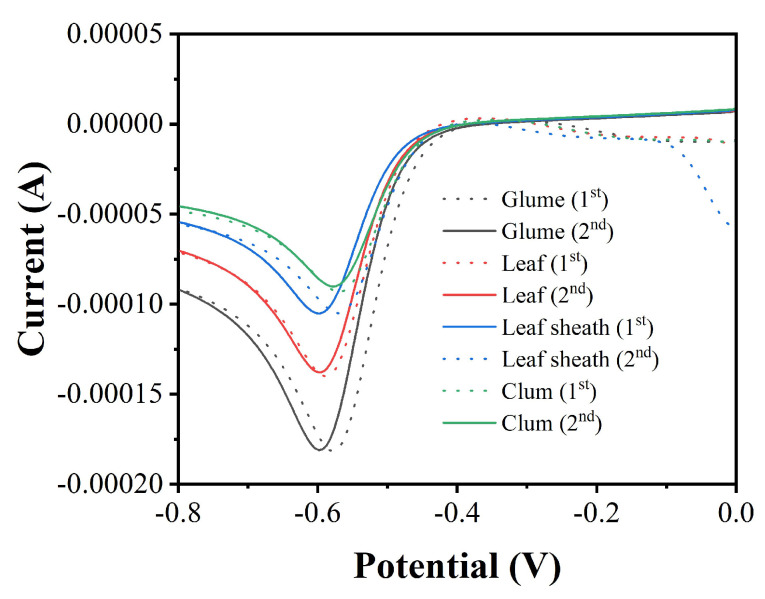
LSV curves of Ag/Si/GCEs prepared using glume, leaf, leaf sheath and culm for electrocatalytic reduction of 0.5 mM H_2_O_2_ in 0.1 M PBS (pH = 7.0).

Figure 4 shows the LSV curves of glume, leaf, leaf sheath and culm samples tested with the proposed electrochemical sensor. As predicted, all LSV curves clearly showed the behavior of electrocatalytic reduction of H_2_O_2_ as the silicon in rice tissue formed silica immobilized on the sensor surface. However, the electrochemical behavior is different from that of the above, with silica immobilized directly on the sensor. These LSV curves have some faint reduction peaks between 0–0.4 V. These reduction peaks may be due to the reduction of some small molecules in plant tissues, such as flavonoids and phenolic acids. These small molecules entered the solvent during the extraction process and were immobilized onto the sensor. Such electrochemical behavior has been observed in our previous studies on plant tissues [42,43,44,45,46]. We performed a second scan with the electrode, and most of these reduction peaks disappeared. At the same time, there was some decrease in the current of electrocatalytic reduction of H_2_O_2_. According to our recent study [47], peroxidase in plant tissues promotes the reduction of H_2_O_2_ by sensors. Therefore, the reduction value of the second LSV scan is more accurate in the actual detection of rice samples. Based on the electrochemical behavior in Figure 4, the highest silicon content was found in rice glume, while the lowest silicon content was found in the culm.

**Figure 5:**
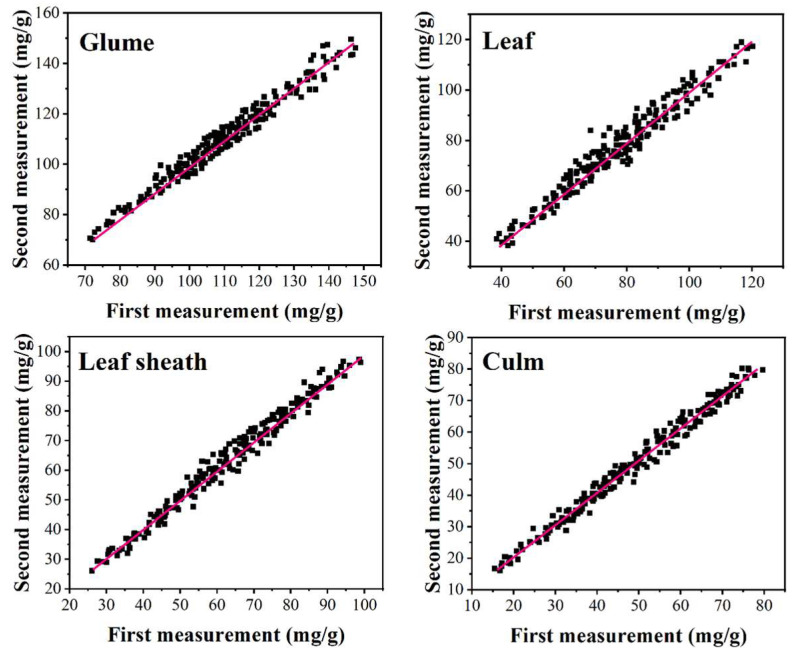
Silicon determination of 200 samples of glume, leaf, leaf sheath and culm.

200 samples of glume, leaf, leaf sheath and culm were tested using the proposed electrochemical sensor. As shown in Figure 5, each sample was tested twice and the results of the two determinations reached a highly significant positive correlation (*p* < 0.01). The linear correlation coefficients for glume, leaf, leaf sheath and culm were 0.9841, 0.9907, 0.9894 and 0.993, respectively. Based on our results above, this novel electrochemical sensor can be used for the detection of silicon content in rice. Furthermore, this electrochemical sensor can also be used for the determination of silicon content in other samples after considering the composition of the sample.

## 4. Conclusions

In summary, this work presented an electrochemical sensor for the detection of silicon content in rice. Silicon in rice tissues was first extracted by HF and formed into silica by pH adjustment. The silica was immobilized onto the sensor surface to become a growth site for Ag nanoparticles and to improve the electrocatalytic performance of the sensor. The amount of silicon can be reflected by the catalytic current through the silica-assisted electrocatalytic reduction of H_2_O_2_. This electrochemical sensor provided a linear detection of 4.67–186.8 mg/g of silicon. We measured the silicon content in rice glume, leaf, leaf sheath and culm and found the highest content in glume and the lowest content in culm. This electrochemical sensor has repeatability and stability comparable to that of conventional spectroscopy. This electrochemical-based sensing strategy is a profiling approach to silicon determination which can be used not only for the determination of silicon content in plants, as using the performance of the catalyst to reflect the amount of inert substrates also provides a new analytical methodology. This methodology can be further extended to the measurement of other electrochemically inert nanoparticles.

## Figures and Tables

**Table 1:** Comparison of proposed electrochemical sensor with silicon-molybdenum blue spectrophotometric analysis.

Si Content (mg)	Detection (mg)		Added (mg)	Detection (mg)		Recovery (%)	
	E^*^	S^*^		E	S	E	S
20	20.17	20.06	10	30.26	30.51	100.87	101.70
40	40.22	40.15	20	61.51	59.04	102.52	98.40
60	58.89	61.20	30	88.83	87.99	98.70	97.77
100	98.57	98.62	40	137.54	142.21	98.24	101.58

E^*^: Electrochemical sensor. S^*^: Spectrophotometric method.

## Supplementary Materials

The following are available online at https://www.mdpi.com/article/10.3390/mi12091048/s1, Figure S1: SEM images of (A) GCE, (B) Si/GCE and (C) Ag/Si/GCE.
