# Supplementary material for: Quantification of Silicon in Rice Based on an Electrochemical Sensor via an Amplified Electrocatalytic Strategy
Source: Micromachines (Basel). 2021 Aug 30;12(9):1048. doi: 10.3390/mi12091048 (PMC8469415; doi:10.3390/mi12091048)
Supplement: Supplementary file 1 [file micromachines-12-01048-s001.zip › micromachines-1323611-supplementary.pdf]

Communication

Quantification of Silicon in Rice Based on an Electrochemical Sensor via an Amplified Electrocatalytic Strategy

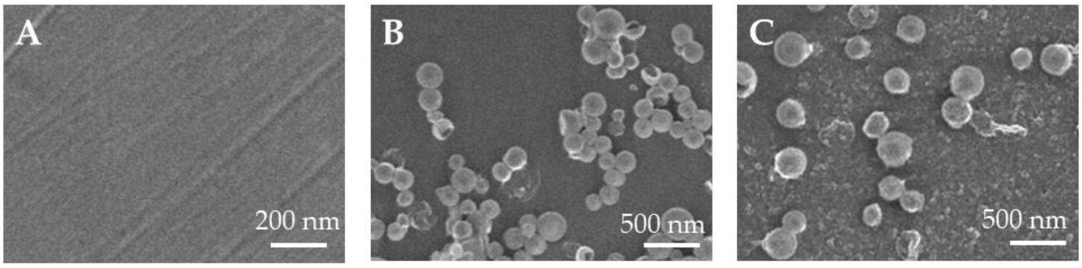

Figure S1. SEM images of (A) GCE, (B) Si/GCE and (C) Ag/Si/GCE.
